# Bis(4-benzoyl-3-methyl-1-phenyl-1*H*-pyrazol-5-olato-κ^2^ *O*,*O*′)bis­(ethanol-κ*O*)cobalt(II)

**DOI:** 10.1107/S1600536812032837

**Published:** 2012-07-25

**Authors:** Omoruyi G. Idemudia, Eric C. Hosten

**Affiliations:** a University of Fort Hare, Department of Chemistry, Private Bag X1314, Alice 5700, South Africa; b Nelson Mandela Metropolitan University, Department of Chemistry, PO Box 77000, Port Elizabeth 6031, South Africa

## Abstract

The title compound, [Co(C_17_H_13_N_2_O_2_)_2_(C_2_H_5_OH)_2_], is a Co^II^ complex with two 4-benzoyl-3-methyl-1-phenyl-1*H*-pyrazol-5-olate (BMPP) ligands and two coordinating ethanol mol­ecules. In the asymmetric unit, there are two half mol­ecules, with the Co^II^ atoms located on inversion centres. The two cobalt complexes have slightly different geometries and in one, the ethyl group of the ethanol is disordered over two sets of sites [occupancy ratio 0.757 (7):0.243 (7)]. Each BMPP ligand is deprotonated with the negative charge delocalized. The hy­droxy group of each ethanol mol­ecule forms hydrogen bonds with a pyrazole N atom in an adjacent BMPP ligand. Weaker C—H⋯O and C—H⋯N inter­actions link the mol­ecules into a three-dimensional structure.

## Related literature

For related structures, see: Raman *et al.* (2001 ▶); Yang *et al.* (2007 ▶). For general background and applications of acyl­pyrazolo­nes, see: Idemudia *et al.* (2012 ▶); Marchetti *et al.* (2005 ▶); Parihar *et al.* (2012 ▶); Zhang *et al.* (2008 ▶).
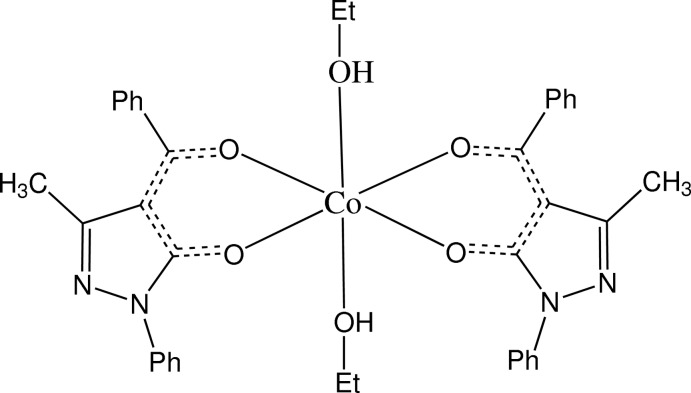


## Experimental

### 

#### Crystal data

[Co(C_17_H_13_N_2_O_2_)_2_(C_2_H_6_O)_2_]
*M* *_r_* = 705.65
Triclinic, 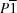

*a* = 11.0484 (3) Å
*b* = 11.2282 (3) Å
*c* = 14.8425 (4) Å
α = 89.205 (1)°
β = 87.678 (1)°
γ = 76.997 (1)°
*V* = 1792.56 (8) Å^3^
*Z* = 2
Mo *K*α radiation
μ = 0.53 mm^−1^
*T* = 200 K
0.49 × 0.36 × 0.12 mm

#### Data collection

Bruker APEXII CCD diffractometer
Absorption correction: numerical (*SADABS*; Bruker, 2008 ▶) *T*_min_ = 0.84, *T*_max_ = 0.94
32307 measured reflections
8869 independent reflections
7482 reflections with *I* > 2σ(*I*)
*R*_int_ = 0.015

#### Refinement

*R*[*F* ^2^ > 2σ(*F* ^2^)] = 0.034
*wR*(*F* ^2^) = 0.095
*S* = 1.02
8869 reflections
471 parameters
H-atom parameters constrained
Δρ_max_ = 0.51 e Å^−3^
Δρ_min_ = −0.47 e Å^−3^

### 

Data collection: *APEX2* (Bruker, 2010 ▶); cell refinement: *SAINT* (Bruker, 2010 ▶); data reduction: *SAINT*; program(s) used to solve structure: *SHELXS97* (Sheldrick, 2008 ▶); program(s) used to refine structure: *SHELXL97* (Sheldrick, 2008 ▶) and *SHELXLE* (Hübschle *et al.*, 2011 ▶); molecular graphics: *ORTEP-3* (Farrugia, 1997 ▶); software used to prepare material for publication: *PLATON* (Spek, 2009 ▶) and *publCIF* (Westrip, 2010 ▶).

## Supplementary Material

Crystal structure: contains datablock(s) global, I. DOI: 10.1107/S1600536812032837/gg2088sup1.cif

Structure factors: contains datablock(s) I. DOI: 10.1107/S1600536812032837/gg2088Isup2.hkl

Supplementary material file. DOI: 10.1107/S1600536812032837/gg2088Isup3.cdx

Additional supplementary materials:  crystallographic information; 3D view; checkCIF report

## Figures and Tables

**Table 1:** Hydrogen-bond geometry (Å, °)

*D*—H⋯*A*	*D*—H	H⋯*A*	*D*⋯*A*	*D*—H⋯*A*
O13—H13*A*⋯N22^i^	0.80	2.04	2.8314 (18)	175
O23—H23*A*⋯N12^ii^	0.89	1.90	2.7862 (16)	177
C16*A*—H16*B*⋯O12	0.99	2.56	3.192 (3)	122
C24—H24*C*⋯O11^iii^	0.98	2.46	3.361 (2)	154
C112—H112⋯O11	0.95	2.24	2.8478 (19)	121
C116—H116⋯O23^iv^	0.95	2.60	3.458 (2)	151
C116—H116⋯N12	0.95	2.50	2.827 (2)	100
C212—H212⋯O21	0.95	2.31	2.848 (2)	115

Symmetry codes: (i) 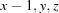; (ii) 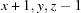; (iii) 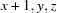; (iv) 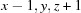.

**Fig. 1.**
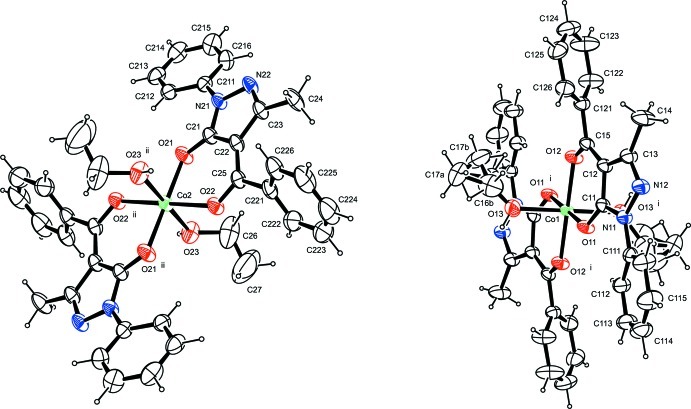
The molecular structure of the title compound (I), with anistropic displacement ellipsoids drawn at the 50% probability level (for clarity not all atom labels are shown. [Symmetry codes: (i) -x, -y, -z + 1; (ii) -x + 2, -y + 1,-z]

**Fig. 2.**
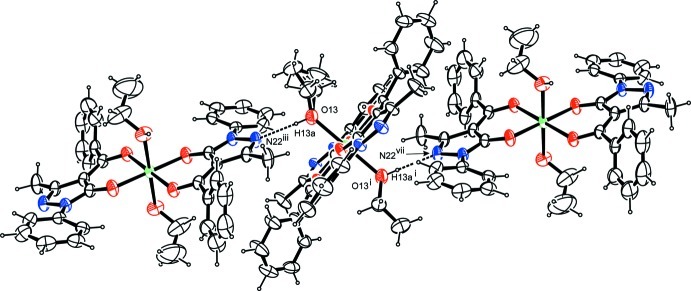
Hydrogen bonding in (I) (anistropic displacements ellipsoids drawn at 50% probability level). [Symmetry codes: (i) -x, -y, -z + 1; (iii) x - 1, y, z; (vii) -x + 1, -y, -z + 1]

**Fig. 3.**
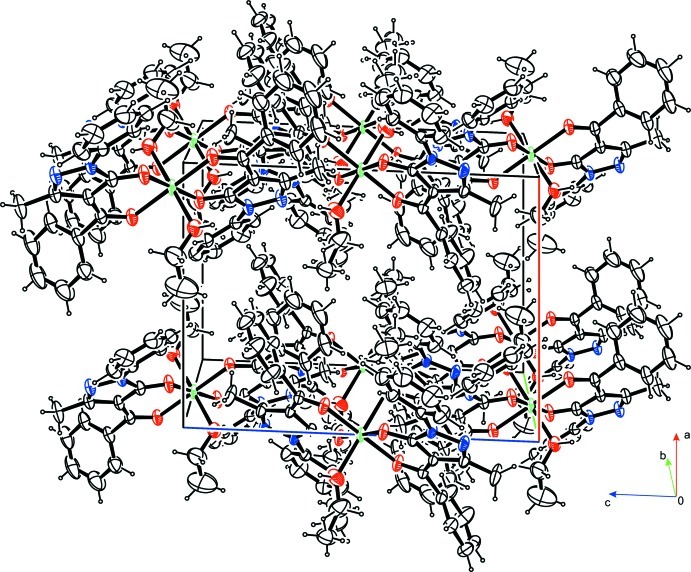
Crystal packing of (I), viewed along [0 1 0] (anistropic displacements ellipsoids drawn at 50% probability level).

## supplementary crystallographic information

### Comment

Acylpyrazolones are excellent spectroscopic chelating agents for the determination of metals in trace amounts (Zhang *et al.*, 2008); they also can be used as heterogeneous catalysts (Parihar *et al.*, 2012). As important β-diketones they react with amino groups to form biologically significant Schiff bases (Idemudia *et al.*, 2012). In our probe on transition metal complexes of Schiff bases, an acylpyrazolone Schiff base was treated with cobalt thiocyanate to obtain the title compound (I), instead of the proposed acylpyrazolone Schiff base cobalt complex. The molecular and crystal structure report is presented herein.

The title compound, C_38_H_38_CoN_4_O_6_, is the Co^II^ complex of two 4-benzoyl-3-methyl-1-phenyl-1*H*-pyrazol-5-olate (BMPP) and two ethanol ligands. There are two half molecules of the complex in the asymmetric unit (Figure 1) with Co located at an inversion centres. The ethanol ethyl groups exhibit disorder: in one complex in a ratio of 0.76:0.24 and to a much lesser extent in the other complex molecule (and hence not modelled).

Each BMPP is deprotonated with the negative charge delocalized over O21···C21···C22···C25···O22 and O11···C11···C12···C15···O12. The average Co—O bond length is 2.042 (39) Å and 2.135 (15) Å for the BMPP and ethanol oxygen atoms respectively. The least squares dihedral angles of the BMPP phenyl groups with the pyrazole ring are 16.61 (9) and 61.27 (9)° for one complex and 24.64 (9) and 62.86 (10)° for the other.

The hydrogen of the hydroxy group of each ethanol is hydrogen bonded to a pyrazole nitrogen in an adjacent BMPP (Figure 2). In this way each complex is hydrogen bonded to four adjacent complex molecules. In addition there are inter and intra molecular contacts and C—H···*Cg*π interactions.

### Experimental

A mixture of 4-benzoylphenylhydrazine-3-methyl-1-phenyl-2-pyrazolin-5-one and cobalt thiocyanate in methanol with a molar ratio of 2:1 respectively, was stirred under reflux for 4 h. The complex molecule of C_38_H_38_CoN_4_O_6_ as orange block-like single crystals and with a melting point 219–220°C suitable for X-ray diffraction analysis was obtained from slow evaporation of the final blue solution at room temperature.

### Refinement

C-bound H atoms were placed in calculated positions and refined as riding atoms, with C—H 0.95 (CH), 0.99 (CH_2_), 0.98 (CH_3_) Å and with *U*_iso_(H)=1.2(1.5 for methyl)*U*_eq_(C). Hydroxy H atoms were located on a Fourier map and allowed to refine freely.

### Crystal data

**Table:**

[Co(C_17_H_13_N_2_O_2_)_2_(C_2_H_6_O)_2_]	*Z* = 2
*M**_r_* = 705.65	*F*(000) = 738
Triclinic, *P*1	*D*_x_ = 1.307 Mg m^−^^3^
Hall symbol: -P 1	Melting point: 219 K
*a* = 11.0484 (3) Å	Mo *K*α radiation, λ = 0.71073 Å
*b* = 11.2282 (3) Å	Cell parameters from 108 reflections
*c* = 14.8425 (4) Å	θ = 3.8–31.5°
α = 89.205 (1)°	µ = 0.53 mm^−^^1^
β = 87.678 (1)°	*T* = 200 K
γ = 76.997 (1)°	Block, orange
*V* = 1792.56 (8) Å^3^	0.49 × 0.36 × 0.12 mm

### Data collection

**Table:**

Bruker APEXII CCD diffractometer	8869 independent reflections
Radiation source: sealed tube	7482 reflections with *I* > 2σ(*I*)
Graphite monochromator	*R*_int_ = 0.015
Detector resolution: 8.3333 pixels mm^-1^	θ_max_ = 28.3°, θ_min_ = 2.3°
φ and ω scans	*h* = −14→14
Absorption correction: numerical (*SADABS*; Bruker, 2008)	*k* = −14→14
*T*_min_ = 0.84, *T*_max_ = 0.94	*l* = −19→19
32307 measured reflections	

### Refinement

**Table:**

Refinement on *F*^2^	Primary atom site location: structure-invariant direct methods
Least-squares matrix: full	Secondary atom site location: difference Fourier map
*R*[*F*^2^ > 2σ(*F*^2^)] = 0.034	Hydrogen site location: inferred from neighbouring sites
*wR*(*F*^2^) = 0.095	H-atom parameters constrained
*S* = 1.02	*w* = 1/[σ^2^(*F*_o_^2^) + (0.0422*P*)^2^ + 0.9543*P*] where *P* = (*F*_o_^2^ + 2*F*_c_^2^)/3
8869 reflections	(Δ/σ)_max_ < 0.001
471 parameters	Δρ_max_ = 0.51 e Å^−^^3^
0 restraints	Δρ_min_ = −0.47 e Å^−^^3^

### Special details

**Table:**

Geometry. All e.s.d.'s (except the e.s.d. in the dihedral angle between two l.s. planes) are estimated using the full covariance matrix. The cell e.s.d.'s are taken into account individually in the estimation of e.s.d.'s in distances, angles and torsion angles; correlations between e.s.d.'s in cell parameters are only used when they are defined by crystal symmetry. An approximate (isotropic) treatment of cell e.s.d.'s is used for estimating e.s.d.'s involving l.s. planes.
Refinement. Refinement of *F*^2^ against ALL reflections. The weighted *R*-factor *wR* and goodness of fit *S* are based on *F*^2^, conventional *R*-factors *R* are based on *F*, with *F* set to zero for negative *F*^2^. The threshold expression of *F*^2^ > σ(*F*^2^) is used only for calculating *R*-factors(gt) *etc*. and is not relevant to the choice of reflections for refinement. *R*-factors based on *F*^2^ are statistically about twice as large as those based on *F*, and *R*- factors based on ALL data will be even larger.

### Fractional atomic coordinates and isotropic or equivalent isotropic displacement parameters (Å^2^)

**Table:**

	*x*	*y*	*z*	*U*_iso_*/*U*_eq_	Occ. (<1)
Co1	0	0	0.5	0.02644 (7)	
Co2	1.0	0.5	0	0.03026 (8)	
O11	−0.06104 (10)	0.16165 (9)	0.56233 (6)	0.0315 (2)	
O12	0.11939 (10)	−0.05987 (10)	0.60361 (7)	0.0336 (2)	
O13	0.14538 (12)	0.06673 (12)	0.43001 (9)	0.0466 (3)	
H13A	0.1259	0.1075	0.3864	0.060 (7)*	
O21	1.07029 (12)	0.34177 (11)	0.06276 (7)	0.0394 (3)	
O22	0.87771 (11)	0.54948 (10)	0.11026 (7)	0.0359 (2)	
O23	0.87494 (12)	0.41461 (12)	−0.06579 (8)	0.0462 (3)	
H23A	0.8985	0.369	−0.1143	0.062 (7)*	
N11	−0.10305 (11)	0.27443 (11)	0.69400 (8)	0.0280 (2)	
N12	−0.05984 (13)	0.26925 (12)	0.78148 (8)	0.0341 (3)	
N21	1.11456 (13)	0.22367 (12)	0.19080 (8)	0.0336 (3)	
N22	1.07617 (14)	0.22544 (13)	0.28176 (8)	0.0375 (3)	
C11	−0.03970 (13)	0.17640 (12)	0.64384 (9)	0.0252 (3)	
C12	0.04931 (14)	0.10603 (13)	0.70179 (9)	0.0279 (3)	
C13	0.02963 (15)	0.17038 (14)	0.78577 (9)	0.0335 (3)	
C14	0.0899 (2)	0.1371 (2)	0.87390 (11)	0.0562 (6)	
H14A	0.0953	0.0502	0.8866	0.084*	
H14B	0.0402	0.1863	0.9221	0.084*	
H14C	0.1736	0.1531	0.8708	0.084*	
C15	0.13206 (13)	−0.00484 (13)	0.67443 (9)	0.0275 (3)	
C21	1.05355 (15)	0.32601 (14)	0.14632 (9)	0.0315 (3)	
C22	0.97286 (15)	0.39911 (14)	0.21254 (9)	0.0320 (3)	
C23	0.99378 (16)	0.32862 (15)	0.29435 (10)	0.0362 (3)	
C24	0.9383 (2)	0.35577 (17)	0.38780 (11)	0.0532 (5)	
H24A	0.8479	0.3844	0.385	0.08*	
H24B	0.9732	0.4192	0.4147	0.08*	
H24C	0.9575	0.2814	0.4247	0.08*	
C25	0.88599 (15)	0.50747 (14)	0.18943 (9)	0.0318 (3)	
C26	0.7822 (3)	0.3650 (3)	−0.01816 (18)	0.0825 (9)	
H26A	0.7871	0.2809	−0.0395	0.099*	
H26B	0.7993	0.3604	0.0469	0.099*	
C27	0.6610 (3)	0.4357 (5)	−0.0300 (4)	0.155 (2)	
H27A	0.6598	0.5222	−0.0209	0.232*	
H27B	0.6032	0.4099	0.0139	0.232*	
H27C	0.6357	0.424	−0.0912	0.232*	
C111	−0.19608 (13)	0.37569 (13)	0.66632 (10)	0.0286 (3)	
C112	−0.25930 (15)	0.37105 (15)	0.58784 (11)	0.0371 (3)	
H112	−0.2418	0.2989	0.5524	0.045*	
C113	−0.34801 (17)	0.47218 (18)	0.56156 (13)	0.0481 (4)	
H113	−0.39	0.4693	0.5073	0.058*	
C114	−0.37636 (19)	0.57684 (18)	0.61289 (15)	0.0535 (5)	
H114	−0.4389	0.6449	0.5952	0.064*	
C115	−0.3129 (2)	0.58125 (18)	0.68994 (16)	0.0578 (5)	
H115	−0.3311	0.6535	0.7252	0.069*	
C116	−0.22273 (18)	0.48182 (16)	0.71716 (13)	0.0463 (4)	
H116	−0.1793	0.4863	0.7705	0.056*	
C121	0.24248 (14)	−0.06123 (14)	0.72766 (9)	0.0309 (3)	
C122	0.2658 (2)	−0.18464 (17)	0.74798 (15)	0.0533 (5)	
H122	0.2096	−0.2321	0.7307	0.064*	
C123	0.3715 (2)	−0.2392 (2)	0.79362 (18)	0.0699 (7)	
H123	0.3862	−0.3235	0.8089	0.084*	
C124	0.45441 (19)	−0.1725 (2)	0.81670 (14)	0.0581 (5)	
H124	0.5263	−0.2104	0.8481	0.07*	
C125	0.43434 (17)	−0.0511 (2)	0.79472 (13)	0.0521 (5)	
H125	0.4936	−0.0055	0.8094	0.063*	
C126	0.32726 (17)	0.00593 (17)	0.75092 (12)	0.0428 (4)	
H12A	0.3125	0.0907	0.737	0.051*	
C211	1.19389 (14)	0.11779 (14)	0.15297 (10)	0.0318 (3)	
C212	1.26245 (15)	0.12585 (17)	0.07298 (10)	0.0382 (3)	
H212	1.2579	0.2026	0.044	0.046*	
C213	1.33730 (16)	0.02042 (19)	0.03630 (12)	0.0463 (4)	
H213	1.3826	0.0252	−0.0189	0.056*	
C214	1.34711 (18)	−0.09108 (19)	0.07852 (14)	0.0516 (5)	
H214	1.3995	−0.1624	0.053	0.062*	
C215	1.28032 (19)	−0.09846 (17)	0.15823 (15)	0.0514 (5)	
H215	1.2875	−0.1751	0.1879	0.062*	
C216	1.20287 (17)	0.00521 (16)	0.19519 (12)	0.0418 (4)	
H216	1.1559	−0.0008	0.2494	0.05*	
C221	0.79264 (16)	0.57662 (15)	0.25689 (10)	0.0375 (3)	
C222	0.66813 (19)	0.5958 (2)	0.23946 (14)	0.0560 (5)	
H222	0.6429	0.5648	0.1858	0.067*	
C223	0.5791 (2)	0.6608 (3)	0.30081 (18)	0.0757 (8)	
H223	0.4931	0.6719	0.2901	0.091*	
C224	0.6170 (3)	0.7090 (2)	0.37703 (16)	0.0757 (8)	
H224	0.5565	0.7535	0.4187	0.091*	
C225	0.7401 (3)	0.69350 (19)	0.39334 (14)	0.0635 (6)	
H225	0.765	0.7285	0.4455	0.076*	
C226	0.8290 (2)	0.62677 (16)	0.33398 (11)	0.0459 (4)	
H226	0.9147	0.6151	0.3458	0.055*	
C16A	0.2742 (3)	−0.0041 (4)	0.4279 (2)	0.0533 (9)	0.757 (7)
H16A	0.2836	−0.0705	0.3832	0.064*	0.757 (7)
H16B	0.2927	−0.0424	0.4878	0.064*	0.757 (7)
C17A	0.3656 (3)	0.0719 (3)	0.4045 (2)	0.0640 (10)	0.757 (7)
H17A	0.45	0.0206	0.4048	0.096*	0.757 (7)
H17B	0.3573	0.1374	0.4488	0.096*	0.757 (7)
H17C	0.3496	0.1078	0.3443	0.096*	0.757 (7)
C16B	0.2583 (12)	0.0716 (13)	0.4401 (8)	0.070 (4)	0.243 (7)
H16C	0.2663	0.1027	0.5012	0.084*	0.243 (7)
H16D	0.2814	0.1302	0.3958	0.084*	0.243 (7)
C17B	0.3450 (12)	−0.0480 (17)	0.4284 (10)	0.106 (6)	0.243 (7)
H17D	0.3669	−0.062	0.3642	0.159*	0.243 (7)
H17E	0.3053	−0.1125	0.4519	0.159*	0.243 (7)
H17F	0.4204	−0.0493	0.4614	0.159*	0.243 (7)

### Atomic displacement parameters (Å^2^)

**Table:**

	*U*^11^	*U*^22^	*U*^33^	*U*^12^	*U*^13^	*U*^23^
Co1	0.03579 (15)	0.02519 (13)	0.01517 (12)	0.00070 (10)	−0.00458 (10)	−0.00412 (9)
Co2	0.04097 (16)	0.03298 (15)	0.01484 (12)	−0.00448 (12)	0.00239 (10)	−0.00391 (10)
O11	0.0441 (6)	0.0282 (5)	0.0180 (4)	0.0022 (4)	−0.0068 (4)	−0.0049 (4)
O12	0.0445 (6)	0.0306 (5)	0.0216 (5)	0.0021 (4)	−0.0091 (4)	−0.0060 (4)
O13	0.0443 (7)	0.0425 (7)	0.0467 (7)	0.0012 (5)	0.0090 (5)	0.0110 (6)
O21	0.0552 (7)	0.0384 (6)	0.0188 (5)	0.0007 (5)	0.0073 (4)	−0.0016 (4)
O22	0.0456 (6)	0.0379 (6)	0.0209 (5)	−0.0031 (5)	0.0045 (4)	−0.0029 (4)
O23	0.0487 (7)	0.0593 (8)	0.0324 (6)	−0.0162 (6)	0.0078 (5)	−0.0235 (5)
N11	0.0322 (6)	0.0298 (6)	0.0199 (5)	−0.0019 (5)	−0.0034 (4)	−0.0068 (4)
N12	0.0400 (7)	0.0393 (7)	0.0200 (5)	−0.0013 (6)	−0.0062 (5)	−0.0098 (5)
N21	0.0415 (7)	0.0373 (7)	0.0195 (5)	−0.0049 (6)	0.0052 (5)	−0.0018 (5)
N22	0.0508 (8)	0.0387 (7)	0.0205 (6)	−0.0065 (6)	0.0079 (5)	−0.0001 (5)
C11	0.0298 (7)	0.0255 (6)	0.0196 (6)	−0.0045 (5)	−0.0013 (5)	−0.0041 (5)
C12	0.0329 (7)	0.0309 (7)	0.0186 (6)	−0.0038 (6)	−0.0047 (5)	−0.0045 (5)
C13	0.0380 (8)	0.0381 (8)	0.0218 (6)	−0.0018 (6)	−0.0063 (6)	−0.0079 (6)
C14	0.0680 (13)	0.0625 (12)	0.0253 (8)	0.0159 (10)	−0.0182 (8)	−0.0149 (8)
C15	0.0324 (7)	0.0293 (7)	0.0199 (6)	−0.0046 (5)	−0.0042 (5)	−0.0005 (5)
C21	0.0389 (8)	0.0337 (7)	0.0217 (6)	−0.0081 (6)	0.0035 (5)	−0.0028 (5)
C22	0.0426 (8)	0.0335 (7)	0.0200 (6)	−0.0094 (6)	0.0062 (6)	−0.0035 (5)
C23	0.0493 (9)	0.0364 (8)	0.0220 (7)	−0.0093 (7)	0.0072 (6)	−0.0013 (6)
C24	0.0847 (15)	0.0415 (9)	0.0252 (8)	−0.0007 (9)	0.0183 (8)	0.0016 (7)
C25	0.0412 (8)	0.0339 (7)	0.0211 (6)	−0.0108 (6)	0.0052 (6)	−0.0051 (5)
C26	0.0781 (17)	0.118 (2)	0.0662 (15)	−0.0562 (17)	0.0244 (13)	−0.0505 (15)
C27	0.066 (2)	0.212 (6)	0.173 (5)	−0.009 (3)	0.028 (3)	−0.017 (4)
C111	0.0271 (7)	0.0295 (7)	0.0274 (7)	−0.0031 (5)	0.0015 (5)	−0.0030 (5)
C112	0.0369 (8)	0.0387 (8)	0.0316 (7)	0.0007 (7)	−0.0040 (6)	−0.0059 (6)
C113	0.0428 (10)	0.0523 (11)	0.0417 (9)	0.0066 (8)	−0.0103 (7)	−0.0007 (8)
C114	0.0479 (11)	0.0429 (10)	0.0596 (12)	0.0119 (8)	−0.0060 (9)	0.0010 (8)
C115	0.0609 (13)	0.0366 (9)	0.0669 (13)	0.0100 (9)	−0.0095 (10)	−0.0157 (9)
C116	0.0484 (10)	0.0384 (9)	0.0476 (10)	0.0022 (7)	−0.0113 (8)	−0.0150 (7)
C121	0.0332 (7)	0.0337 (7)	0.0232 (6)	−0.0008 (6)	−0.0048 (5)	−0.0050 (5)
C122	0.0572 (12)	0.0383 (9)	0.0645 (12)	−0.0062 (8)	−0.0304 (10)	0.0048 (8)
C123	0.0739 (15)	0.0438 (11)	0.0865 (17)	0.0045 (10)	−0.0421 (13)	0.0066 (11)
C124	0.0432 (10)	0.0688 (14)	0.0531 (11)	0.0107 (9)	−0.0203 (9)	−0.0118 (10)
C125	0.0369 (9)	0.0748 (14)	0.0462 (10)	−0.0138 (9)	−0.0103 (8)	−0.0100 (9)
C126	0.0444 (9)	0.0461 (10)	0.0392 (9)	−0.0117 (8)	−0.0092 (7)	−0.0016 (7)
C211	0.0302 (7)	0.0381 (8)	0.0268 (7)	−0.0072 (6)	0.0002 (5)	−0.0065 (6)
C212	0.0333 (8)	0.0508 (10)	0.0278 (7)	−0.0039 (7)	−0.0001 (6)	−0.0014 (6)
C213	0.0337 (8)	0.0670 (12)	0.0339 (8)	−0.0023 (8)	0.0031 (6)	−0.0119 (8)
C214	0.0435 (10)	0.0516 (11)	0.0573 (11)	−0.0056 (8)	0.0050 (8)	−0.0246 (9)
C215	0.0536 (11)	0.0357 (9)	0.0656 (12)	−0.0125 (8)	0.0071 (9)	−0.0110 (8)
C216	0.0468 (10)	0.0374 (9)	0.0424 (9)	−0.0138 (7)	0.0093 (7)	−0.0062 (7)
C221	0.0493 (9)	0.0329 (8)	0.0263 (7)	−0.0035 (7)	0.0128 (6)	0.0001 (6)
C222	0.0509 (11)	0.0668 (13)	0.0429 (10)	0.0005 (10)	0.0082 (8)	0.0024 (9)
C223	0.0549 (13)	0.0874 (18)	0.0668 (15)	0.0166 (12)	0.0210 (11)	0.0144 (13)
C224	0.095 (2)	0.0621 (14)	0.0505 (13)	0.0167 (13)	0.0379 (13)	0.0018 (10)
C225	0.0972 (18)	0.0469 (11)	0.0376 (10)	−0.0019 (11)	0.0259 (11)	−0.0102 (8)
C226	0.0682 (12)	0.0375 (9)	0.0301 (8)	−0.0107 (8)	0.0154 (8)	−0.0065 (6)
C16A	0.0383 (16)	0.055 (2)	0.0609 (17)	0.0003 (14)	0.0033 (12)	0.0128 (14)
C17A	0.0478 (17)	0.072 (2)	0.073 (2)	−0.0154 (14)	0.0098 (14)	−0.0167 (16)
C16B	0.083 (8)	0.059 (7)	0.083 (7)	−0.043 (6)	−0.025 (6)	0.007 (6)
C17B	0.047 (7)	0.152 (15)	0.098 (10)	0.022 (8)	−0.013 (6)	0.011 (9)

### Geometric parameters (Å, º)

**Table:**

Co1—O11	2.0125 (10)	C112—H112	0.95
Co1—O11^i^	2.0125 (10)	C113—C114	1.378 (3)
Co1—O12	2.0721 (10)	C113—H113	0.95
Co1—O12^i^	2.0721 (10)	C114—C115	1.372 (3)
Co1—O13	2.1460 (12)	C114—H114	0.95
Co1—O13^i^	2.1461 (12)	C115—C116	1.387 (3)
Co2—O21	2.0047 (11)	C115—H115	0.95
Co2—O21^ii^	2.0047 (11)	C116—H116	0.95
Co2—O22	2.0786 (10)	C121—C122	1.382 (2)
Co2—O22^ii^	2.0786 (10)	C121—C126	1.384 (2)
Co2—O23	2.1245 (12)	C122—C123	1.389 (3)
Co2—O23^ii^	2.1245 (12)	C122—H122	0.95
O11—C11	1.2622 (16)	C123—C124	1.363 (3)
O12—C15	1.2550 (16)	C123—H123	0.95
O13—C16B	1.276 (11)	C124—C125	1.368 (3)
O13—C16A	1.465 (3)	C124—H124	0.95
O13—H13A	0.7956	C125—C126	1.394 (3)
O21—C21	1.2624 (17)	C125—H125	0.95
O22—C25	1.2593 (18)	C126—H12A	0.95
O23—C26	1.431 (3)	C211—C216	1.388 (2)
O23—H23A	0.8858	C211—C212	1.395 (2)
N11—C11	1.3746 (17)	C212—C213	1.386 (2)
N11—N12	1.3974 (16)	C212—H212	0.95
N11—C111	1.4189 (18)	C213—C214	1.376 (3)
N12—C13	1.313 (2)	C213—H213	0.95
N21—C21	1.370 (2)	C214—C215	1.380 (3)
N21—N22	1.3976 (16)	C214—H214	0.95
N21—C211	1.4165 (19)	C215—C216	1.385 (2)
N22—C23	1.313 (2)	C215—H215	0.95
C11—C12	1.4252 (19)	C216—H216	0.95
C12—C15	1.422 (2)	C221—C222	1.378 (3)
C12—C13	1.4336 (18)	C221—C226	1.394 (2)
C13—C14	1.496 (2)	C222—C223	1.397 (3)
C14—H14A	0.98	C222—H222	0.95
C14—H14B	0.98	C223—C224	1.380 (4)
C14—H14C	0.98	C223—H223	0.95
C15—C121	1.4934 (19)	C224—C225	1.363 (4)
C21—C22	1.434 (2)	C224—H224	0.95
C22—C25	1.417 (2)	C225—C226	1.385 (3)
C22—C23	1.438 (2)	C225—H225	0.95
C23—C24	1.500 (2)	C226—H226	0.95
C24—H24A	0.98	C16A—C17A	1.491 (5)
C24—H24B	0.98	C16A—H16A	0.99
C24—H24C	0.98	C16A—H16B	0.99
C25—C221	1.499 (2)	C17A—H17A	0.98
C26—C27	1.411 (5)	C17A—H17B	0.98
C26—H26A	0.99	C17A—H17C	0.98
C26—H26B	0.99	C16B—C17B	1.47 (2)
C27—H27A	0.98	C16B—H16C	0.99
C27—H27B	0.98	C16B—H16D	0.99
C27—H27C	0.98	C17B—H17D	0.98
C111—C116	1.387 (2)	C17B—H17E	0.98
C111—C112	1.390 (2)	C17B—H17F	0.98
C112—C113	1.386 (2)		
			
O11—Co1—O11^i^	180.0	H27A—C27—H27B	109.5
O11—Co1—O12	90.12 (4)	C26—C27—H27C	109.5
O11^i^—Co1—O12	89.88 (4)	H27A—C27—H27C	109.5
O11—Co1—O12^i^	89.88 (4)	H27B—C27—H27C	109.5
O11^i^—Co1—O12^i^	90.12 (4)	C116—C111—C112	119.39 (15)
O12—Co1—O12^i^	180.0	C116—C111—N11	119.69 (14)
O11—Co1—O13	90.93 (5)	C112—C111—N11	120.91 (13)
O11^i^—Co1—O13	89.07 (5)	C113—C112—C111	119.63 (15)
O12—Co1—O13	89.14 (5)	C113—C112—H112	120.2
O12^i^—Co1—O13	90.86 (5)	C111—C112—H112	120.2
O11—Co1—O13^i^	89.07 (5)	C114—C113—C112	121.06 (17)
O11^i^—Co1—O13^i^	90.93 (5)	C114—C113—H113	119.5
O12—Co1—O13^i^	90.86 (5)	C112—C113—H113	119.5
O12^i^—Co1—O13^i^	89.14 (5)	C115—C114—C113	119.04 (17)
O13—Co1—O13^i^	180.0	C115—C114—H114	120.5
O21—Co2—O21^ii^	180.0	C113—C114—H114	120.5
O21—Co2—O22	88.73 (4)	C114—C115—C116	121.02 (18)
O21^ii^—Co2—O22	91.27 (4)	C114—C115—H115	119.5
O21—Co2—O22^ii^	91.27 (4)	C116—C115—H115	119.5
O21^ii^—Co2—O22^ii^	88.73 (4)	C115—C116—C111	119.84 (17)
O22—Co2—O22^ii^	179.9980 (10)	C115—C116—H116	120.1
O21—Co2—O23	89.54 (5)	C111—C116—H116	120.1
O21^ii^—Co2—O23	90.46 (5)	C122—C121—C126	119.27 (15)
O22—Co2—O23	92.76 (4)	C122—C121—C15	119.56 (14)
O22^ii^—Co2—O23	87.24 (4)	C126—C121—C15	120.97 (14)
O21—Co2—O23^ii^	90.46 (5)	C121—C122—C123	120.02 (19)
O21^ii^—Co2—O23^ii^	89.54 (5)	C121—C122—H122	120.0
O22—Co2—O23^ii^	87.24 (4)	C123—C122—H122	120.0
O22^ii^—Co2—O23^ii^	92.76 (4)	C124—C123—C122	120.4 (2)
O23—Co2—O23^ii^	180.0	C124—C123—H123	119.8
C11—O11—Co1	121.85 (9)	C122—C123—H123	119.8
C15—O12—Co1	128.43 (9)	C123—C124—C125	120.19 (18)
C16B—O13—Co1	139.3 (5)	C123—C124—H124	119.9
C16A—O13—Co1	121.07 (15)	C125—C124—H124	119.9
C16B—O13—H13A	104.2	C124—C125—C126	120.19 (18)
C16A—O13—H13A	115.2	C124—C125—H125	119.9
Co1—O13—H13A	116.1	C126—C125—H125	119.9
C21—O21—Co2	122.73 (10)	C121—C126—C125	119.86 (18)
C25—O22—Co2	128.58 (10)	C121—C126—H12A	120.1
C26—O23—Co2	122.93 (12)	C125—C126—H12A	120.1
C26—O23—H23A	106.9	C216—C211—C212	119.73 (15)
Co2—O23—H23A	121.5	C216—C211—N21	119.95 (14)
C11—N11—N12	111.13 (11)	C212—C211—N21	120.32 (14)
C11—N11—C111	128.62 (11)	C213—C212—C211	119.18 (17)
N12—N11—C111	120.16 (11)	C213—C212—H212	120.4
C13—N12—N11	106.47 (11)	C211—C212—H212	120.4
C21—N21—N22	111.42 (12)	C214—C213—C212	121.13 (17)
C21—N21—C211	127.65 (12)	C214—C213—H213	119.4
N22—N21—C211	120.37 (12)	C212—C213—H213	119.4
C23—N22—N21	106.32 (12)	C213—C214—C215	119.52 (17)
O11—C11—N11	122.94 (12)	C213—C214—H214	120.2
O11—C11—C12	131.05 (13)	C215—C214—H214	120.2
N11—C11—C12	105.98 (11)	C214—C215—C216	120.40 (19)
C15—C12—C11	122.89 (12)	C214—C215—H215	119.8
C15—C12—C13	132.25 (13)	C216—C215—H215	119.8
C11—C12—C13	104.83 (12)	C215—C216—C211	120.02 (16)
N12—C13—C12	111.57 (12)	C215—C216—H216	120.0
N12—C13—C14	118.60 (13)	C211—C216—H216	120.0
C12—C13—C14	129.73 (14)	C222—C221—C226	119.56 (17)
C13—C14—H14A	109.5	C222—C221—C25	118.70 (16)
C13—C14—H14B	109.5	C226—C221—C25	121.67 (16)
H14A—C14—H14B	109.5	C221—C222—C223	119.9 (2)
C13—C14—H14C	109.5	C221—C222—H222	120.0
H14A—C14—H14C	109.5	C223—C222—H222	120.0
H14B—C14—H14C	109.5	C224—C223—C222	119.5 (2)
O12—C15—C12	122.74 (12)	C224—C223—H223	120.2
O12—C15—C121	115.73 (13)	C222—C223—H223	120.2
C12—C15—C121	121.50 (12)	C225—C224—C223	120.8 (2)
O21—C21—N21	122.37 (13)	C225—C224—H224	119.6
O21—C21—C22	131.49 (15)	C223—C224—H224	119.6
N21—C21—C22	106.11 (12)	C224—C225—C226	120.0 (2)
C25—C22—C21	122.18 (13)	C224—C225—H225	120.0
C25—C22—C23	133.13 (14)	C226—C225—H225	120.0
C21—C22—C23	104.25 (13)	C225—C226—C221	120.1 (2)
N22—C23—C22	111.88 (13)	C225—C226—H226	120.0
N22—C23—C24	117.70 (14)	C221—C226—H226	120.0
C22—C23—C24	130.42 (15)	O13—C16A—C17A	112.7 (3)
C23—C24—H24A	109.5	O13—C16A—H16A	109.1
C23—C24—H24B	109.5	C17A—C16A—H16A	109.1
H24A—C24—H24B	109.5	O13—C16A—H16B	109.1
C23—C24—H24C	109.5	C17A—C16A—H16B	109.1
H24A—C24—H24C	109.5	H16A—C16A—H16B	107.8
H24B—C24—H24C	109.5	O13—C16B—C17B	112.6 (11)
O22—C25—C22	122.65 (13)	O13—C16B—H16C	109.1
O22—C25—C221	115.15 (14)	C17B—C16B—H16C	109.1
C22—C25—C221	122.12 (13)	O13—C16B—H16D	109.1
C27—C26—O23	112.4 (3)	C17B—C16B—H16D	109.1
C27—C26—H26A	109.1	H16C—C16B—H16D	107.8
O23—C26—H26A	109.1	C16B—C17B—H17D	109.5
C27—C26—H26B	109.1	C16B—C17B—H17E	109.5
O23—C26—H26B	109.1	H17D—C17B—H17E	109.5
H26A—C26—H26B	107.9	C16B—C17B—H17F	109.5
C26—C27—H27A	109.5	H17D—C17B—H17F	109.5
C26—C27—H27B	109.5	H17E—C17B—H17F	109.5
			
O12—Co1—O11—C11	−14.75 (12)	N21—N22—C23—C24	−178.81 (16)
O12^i^—Co1—O11—C11	165.25 (12)	C25—C22—C23—N22	172.28 (17)
O13—Co1—O11—C11	−103.89 (12)	C21—C22—C23—N22	0.03 (19)
O13^i^—Co1—O11—C11	76.11 (12)	C25—C22—C23—C24	−8.4 (3)
O11—Co1—O12—C15	2.27 (13)	C21—C22—C23—C24	179.35 (19)
O11^i^—Co1—O12—C15	−177.73 (13)	Co2—O22—C25—C22	−12.6 (2)
O13—Co1—O12—C15	93.20 (13)	Co2—O22—C25—C221	170.58 (11)
O13^i^—Co1—O12—C15	−86.80 (13)	C21—C22—C25—O22	−3.2 (2)
O11—Co1—O13—C16B	90.8 (10)	C23—C22—C25—O22	−174.33 (17)
O11^i^—Co1—O13—C16B	−89.2 (10)	C21—C22—C25—C221	173.43 (15)
O12—Co1—O13—C16B	0.7 (10)	C23—C22—C25—C221	2.3 (3)
O12^i^—Co1—O13—C16B	−179.3 (10)	Co2—O23—C26—C27	108.5 (3)
O11—Co1—O13—C16A	131.2 (2)	C11—N11—C111—C116	−160.85 (16)
O11^i^—Co1—O13—C16A	−48.8 (2)	N12—N11—C111—C116	15.4 (2)
O12—Co1—O13—C16A	41.1 (2)	C11—N11—C111—C112	17.8 (2)
O12^i^—Co1—O13—C16A	−138.9 (2)	N12—N11—C111—C112	−165.92 (14)
O22—Co2—O21—C21	−17.43 (13)	C116—C111—C112—C113	−0.1 (3)
O22^ii^—Co2—O21—C21	162.58 (13)	N11—C111—C112—C113	−178.76 (16)
O23—Co2—O21—C21	−110.20 (13)	C111—C112—C113—C114	−1.2 (3)
O23^ii^—Co2—O21—C21	69.80 (13)	C112—C113—C114—C115	1.7 (3)
O21—Co2—O22—C25	19.90 (13)	C113—C114—C115—C116	−0.9 (4)
O21^ii^—Co2—O22—C25	−160.10 (13)	C114—C115—C116—C111	−0.3 (3)
O23—Co2—O22—C25	109.38 (13)	C112—C111—C116—C115	0.8 (3)
O23^ii^—Co2—O22—C25	−70.62 (13)	N11—C111—C116—C115	179.53 (18)
O21—Co2—O23—C26	62.54 (19)	O12—C15—C121—C122	−49.2 (2)
O21^ii^—Co2—O23—C26	−117.46 (19)	C12—C15—C121—C122	132.44 (18)
O22—Co2—O23—C26	−26.17 (19)	O12—C15—C121—C126	125.62 (16)
O22^ii^—Co2—O23—C26	153.83 (19)	C12—C15—C121—C126	−52.7 (2)
C11—N11—N12—C13	0.52 (17)	C126—C121—C122—C123	1.9 (3)
C111—N11—N12—C13	−176.35 (14)	C15—C121—C122—C123	176.9 (2)
C21—N21—N22—C23	−1.08 (18)	C121—C122—C123—C124	−1.6 (4)
C211—N21—N22—C23	−173.12 (14)	C122—C123—C124—C125	−0.3 (4)
Co1—O11—C11—N11	−164.27 (10)	C123—C124—C125—C126	1.9 (3)
Co1—O11—C11—C12	17.8 (2)	C122—C121—C126—C125	−0.3 (3)
N12—N11—C11—O11	−179.12 (13)	C15—C121—C126—C125	−175.20 (16)
C111—N11—C11—O11	−2.6 (2)	C124—C125—C126—C121	−1.6 (3)
N12—N11—C11—C12	−0.73 (16)	C21—N21—C211—C216	−150.36 (17)
C111—N11—C11—C12	175.81 (14)	N22—N21—C211—C216	20.3 (2)
O11—C11—C12—C15	−2.9 (3)	C21—N21—C211—C212	29.1 (2)
N11—C11—C12—C15	178.94 (13)	N22—N21—C211—C212	−160.23 (15)
O11—C11—C12—C13	178.84 (16)	C216—C211—C212—C213	0.9 (2)
N11—C11—C12—C13	0.64 (16)	N21—C211—C212—C213	−178.58 (15)
N11—N12—C13—C12	−0.09 (18)	C211—C212—C213—C214	−1.5 (3)
N11—N12—C13—C14	−176.89 (16)	C212—C213—C214—C215	0.7 (3)
C15—C12—C13—N12	−178.42 (16)	C213—C214—C215—C216	0.7 (3)
C11—C12—C13—N12	−0.35 (18)	C214—C215—C216—C211	−1.2 (3)
C15—C12—C13—C14	−2.1 (3)	C212—C211—C216—C215	0.4 (3)
C11—C12—C13—C14	176.00 (19)	N21—C211—C216—C215	179.93 (16)
Co1—O12—C15—C12	9.8 (2)	O22—C25—C221—C222	54.7 (2)
Co1—O12—C15—C121	−168.49 (10)	C22—C25—C221—C222	−122.18 (19)
C11—C12—C15—O12	−12.5 (2)	O22—C25—C221—C226	−122.06 (17)
C13—C12—C15—O12	165.27 (16)	C22—C25—C221—C226	61.1 (2)
C11—C12—C15—C121	165.69 (14)	C226—C221—C222—C223	−2.6 (3)
C13—C12—C15—C121	−16.5 (3)	C25—C221—C222—C223	−179.47 (19)
Co2—O21—C21—N21	−171.43 (11)	C221—C222—C223—C224	2.2 (4)
Co2—O21—C21—C22	10.5 (3)	C222—C223—C224—C225	−0.2 (4)
N22—N21—C21—O21	−177.42 (15)	C223—C224—C225—C226	−1.3 (4)
C211—N21—C21—O21	−6.1 (3)	C224—C225—C226—C221	0.8 (3)
N22—N21—C21—C22	1.09 (18)	C222—C221—C226—C225	1.2 (3)
C211—N21—C21—C22	172.42 (15)	C25—C221—C226—C225	177.89 (17)
O21—C21—C22—C25	4.3 (3)	C16B—O13—C16A—C17A	−27.7 (10)
N21—C21—C22—C25	−173.99 (14)	Co1—O13—C16A—C17A	−160.64 (19)
O21—C21—C22—C23	177.64 (17)	C16A—O13—C16B—C17B	−1.5 (7)
N21—C21—C22—C23	−0.67 (17)	Co1—O13—C16B—C17B	72.6 (14)
N21—N22—C23—C22	0.61 (19)		

Symmetry codes: (i) −*x*, −*y*, −*z*+1; (ii) −*x*+2, −*y*+1, −*z*.

### Hydrogen-bond geometry (Å, º)

**Table:**

*D*—H···*A*	*D*—H	H···*A*	*D*···*A*	*D*—H···*A*
O13—H13*A*···N22^iii^	0.80	2.04	2.8314 (18)	175
O23—H23*A*···N12^iv^	0.89	1.90	2.7862 (16)	177
C16*A*—H16*B*···O12	0.99	2.56	3.192 (3)	122
C24—H24*C*···O11^v^	0.98	2.46	3.361 (2)	154
C112—H112···O11	0.95	2.24	2.8478 (19)	121
C116—H116···O23^vi^	0.95	2.60	3.458 (2)	151
C116—H116···N12	0.95	2.50	2.827 (2)	100
C212—H212···O21	0.95	2.31	2.848 (2)	115

Symmetry codes: (iii) *x*−1, *y*, *z*; (iv) *x*+1, *y*, *z*−1; (v) *x*+1, *y*, *z*; (vi) *x*−1, *y*, *z*+1.
